# Physiologically based pharmacokinetic modelling to predict the clinical effect of CYP3A inhibitors/inducers on esaxerenone pharmacokinetics in healthy subjects and subjects with hepatic impairment

**DOI:** 10.1007/s00228-021-03194-x

**Published:** 2021-08-20

**Authors:** Akiko Watanabe, Tomoko Ishizuka, Makiko Yamada, Yoshiyuki Igawa, Takako Shimizu, Hitoshi Ishizuka

**Affiliations:** 1 Quantitative Clinical Pharmacology Department, Daiichi Sankyo Co., Ltd., Tokyo, Japan; 2 Drug Metabolism and Pharmacokinetics Research Laboratories, Daiichi Sankyo Co., Ltd., Tokyo, Japan

**Keywords:** CYP3A inhibitors/inducers, Drug–drug interactions, Esaxerenone, Hepatic impairment, Physiologically based pharmacokinetics

## Abstract

**Purpose:**

Esaxerenone is a novel, oral, nonsteroidal treatment for hypertension. Physiologically based pharmacokinetic (PBPK) modelling was performed to predict the drug–drug interaction (DDI) effect of cytochrome P450 (CYP)3A modulators on esaxerenone pharmacokinetics in healthy subjects and subjects with hepatic impairment.

**Methods:**

In our PBPK model, the fraction of esaxerenone metabolised by CYP3A was estimated from mass-balance data and verified and optimised by clinical DDI study results with strong CYP3A modulators. The model was also verified by the observed pharmacokinetics after multiple oral dosing and by the effect of hepatic impairment on esaxerenone pharmacokinetics. The model was applied to predict the DDI effects on esaxerenone pharmacokinetics with untested CYP3A modulators in healthy subjects and with strong CYP3A modulators in subjects with hepatic impairment.

**Results:**

The PBPK model well described esaxerenone pharmacokinetics after multiple oral dosing. The predicted fold changes in esaxerenone plasma exposure after coadministration with strong CYP3A modulators were comparable with the observed data (1.53-fold with itraconazole and 0.31-fold with rifampicin). Predicted DDIs with untested moderate CYP3A modulators were less than the observed DDI with strong CYP3A modulators. The PBPK model also described the effect of hepatic impairment on esaxerenone plasma exposure. The predicted DDI results with strong CYP3A modulators in subjects with hepatic impairment indicate that, for concomitant use of CYP3A modulators, caution is advised for subjects with hepatic impairment, as is for healthy subjects.

**Conclusion:**

The PBPK model developed predicted esaxerenone pharmacokinetics and DDIs and informed concurrent use of esaxerenone with CYP3A modulators.

**Supplementary information:**

The online version contains supplementary material available at 10.1007/s00228-021-03194-x.

## Introduction

Esaxerenone (CS-3150) is a novel, oral, nonsteroidal, selective mineralocorticoid receptor blocker [1]. Its chemical structure is shown in Online Resource 1. In January 2019, esaxerenone was approved in Japan for the treatment of hypertension, following a successful phase III trial in Japanese patients with essential hypertension [2, 3], and ascertainment of a dose-proportional pharmacokinetic (PK) profile in a double-blinded phase I, sequential, dose-escalation study in healthy Japanese subjects [4]. Following a single oral dose (range 5–200 mg) of esaxerenone, the time to reach maximum plasma concentration (*t*_max_) was 2.5–3.5 h, and the elimination half-life (*t*_1/2_) was 18.7–22.9 h. Similar results were observed following multiple dosing of esaxerenone (10–100 mg/day for 10 days), with a *t*_max_ range of 2.5–3.5 h and a *t*_1/2_ range of 22.3–25.1 h. The steady state was reached at day 4, and the mean observed accumulation ratio ranged from 1.36 (10 mg dose) to 1.98 (20 mg dose). Regarding the clinical efficacy for essential hypertension, esaxerenone 2.5 mg/day was shown to be noninferior to eplerenone 50 mg/day. Furthermore, the esaxerenone 5 mg/day dose was superior to the 2.5 mg/day dose. Both doses of esaxerenone demonstrated efficacy in lowering blood pressure and were well tolerated. However, hyperkalaemia has long been recognized as a potential side effect that occurs during treatment with mineralocorticoid receptor blockers [5]. The approved dosing regimen of esaxerenone for hypertension is to initiate treatment at 2.5 mg once daily and titrate to a maximum of 5 mg once daily within 4 weeks when the effect is insufficient [2]. Dose adjustments may be required based on potassium levels [2].

Esaxerenone exhibited good oral absorption (90% bioavailability) in an absolute bioavailability study [6] and demonstrated a clinically favourable absorption, distribution, metabolism, and excretion profile in mass-balance studies [7]. Moreover, esaxerenone is a substrate of cytochrome P450 (CYP)3A, as well as multiple uridine 5′-diphospho-glucuronosyltransferase (UGT) isoforms, with oxidation by CYP3A contributing to approximately 30% of esaxerenone clearance. Esaxerenone is eliminated via multiple pathways including oxidation, glucuronidation, and hydrolysis; however, urinary excretion is low [7].

Esaxerenone is also a substrate of P-glycoprotein (P-gp) and breast cancer resistance protein (BCRP); however, the drug–drug interaction (DDI) risks with P-gp/BCRP inhibitors are expected to be low because of high oral absorption. In addition, in vitro studies with pooled human liver microsomes and primary cultures of human hepatocytes showed that esaxerenone inhibited CYP3A in both a competitive and time-dependent manner and induced CYP3A activity [8].

CYP3A inhibitors and inducers have been used to investigate the potential victim DDI risks of esaxerenone. Itraconazole, a strong CYP3A inhibitor, increased the area under the curve (AUC) by 1.5 times when co-administered with esaxerenone, while rifampicin, a strong CYP3A inducer, reduced the AUC by a third and shortened the *t*_1/2_; thus, considerations should be taken when administering esaxerenone alongside both strong CYP3A inhibitors and inducers [9].

Hepatic impairment reduces drug elimination and alters the PK profile of some drugs; therefore, dose adjustments are needed for patients with altered hepatic function. A multicentre, single-arm, open-label, parallel-group study in subjects with mild and moderate hepatic impairment showed no difference in the PK profile in subjects administered a single oral 2.5 mg dose of esaxerenone 30 min after a standard meal compared with the normal hepatic function group, indicating no requirement for dose adjustments in this subject group [10].

The investigation of DDIs for anti-hypertensive drugs is important because these long-term treatments are likely to be used in combination with drugs that have CYP modulating effects. Furthermore, most hypertensive patients require multiple concurrent treatments to achieve target blood pressure. Triple therapies are common and generally consist of a renin–angiotensin system inhibitor, a calcium channel blocker, and a diuretic. The addition of a mineralocorticoid receptor blocker is also recommended in the Japanese Guidelines for the Management of Hypertension (2019) for the treatment of poorly controlled blood pressure or resistant hypertension [11].

The presence and magnitude of DDIs can be predicted via physiologically based pharmacokinetic (PBPK) modelling, which enables the simulation of untested clinical scenarios by integrating physiological, chemical, and drug-dependent preclinical and clinical information. The use of PBPK analysis in regulatory submissions to the US Food and Drug Administration [12], European Medicines Agency [13], and Pharmaceuticals and Medical Devices Agency [14] has increased in recent years, primarily to identify DDIs. The use of PBPK modelling to identify dosing regimens for specific populations, including those with renal and hepatic impairment, has also increased [12–14]. The implementation of PBPK modelling has enabled the production of dosage and safety guidelines in lieu of extensive clinical trials [14]. The objective of this study was to evaluate the effects of untested CYP3A inhibitors and inducers on esaxerenone PK by means of PBPK modelling in both healthy subjects and in subjects with hepatic impairment.

## Methods

### Clinical pharmacokinetic data

Absolute bioavailability data used to build the PBPK model were obtained from an open-label crossover study of healthy Japanese subjects after a single oral dose (5 mg) of esaxerenone [6]. Absolute bioavailability was determined as 89.0% in the fasting state and 90.8% postprandially. The following PK parameters were also obtained from this study: steady-state volume of distribution (*V*_*ss*_, 1.27 L/kg) and intravenous clearance (CL_iv_, 0.97 mL/min/kg) [6].

Mass-balance data were obtained from a single oral dose (20 mg) administration of [^14^C]-esaxerenone in healthy subjects [7]. Oxidation by CYP3A was estimated to contribute approximately 30% to the clearance. Multiple-dosing PK data were obtained from a DDI study with midazolam and oral esaxerenone (5 mg), administered once daily for 14 days [15].

An open-label, single-sequence, crossover study conducted in healthy Japanese subjects provided PK data for DDIs with CYP3A inhibitors and inducers (itraconazole and rifampicin, respectively) [9]. Each subject received an oral dose of esaxerenone (2.5 mg) on day 1 (single-dose administration phase), followed by oral itraconazole (200 mg twice daily on day 8 and once daily from day 9 to 16), in conjunction with a second oral dose of esaxerenone (2.5 mg) on day 13 (coadministration phase). A similar study design was conducted with rifampicin, with each subject administered an oral dose of esaxerenone (5 mg) on days 1 and 13, with oral rifampicin (600 mg) co-administered once daily on days 8 to 16 [9].

PK data for subjects with hepatic impairment were obtained from a single-dose PK and safety study in Japanese subjects with mild to moderate (Child–Pugh grade A [CP-A] or B [CP-B]) hepatic impairment, and healthy controls with normal hepatic function matched by age. Each subject received a single 2.5 mg oral dose of esaxerenone [10].

### PBPK modelling and simulation

For all PBPK modelling and simulations described, population-based PBPK software (Simcyp^®^, v17.0.0, Simcyp Ltd, Sheffield, UK) was used. Trial designs for simulations are listed in Online Resource 2, and the number of subjects per trial was the same as in the source clinical studies. The software’s built-in files for inhibitor/inducer and population were used without modification, except for the distribution of ages in the population file of healthy subjects (Sim-Healthy Volunteers), which was modified to match the distribution of ages in subjects with hepatic impairment (Sim-Cirrhosis CP-A and CP-B) for PK simulation in subjects with normal hepatic function.

**Fig. 1:**
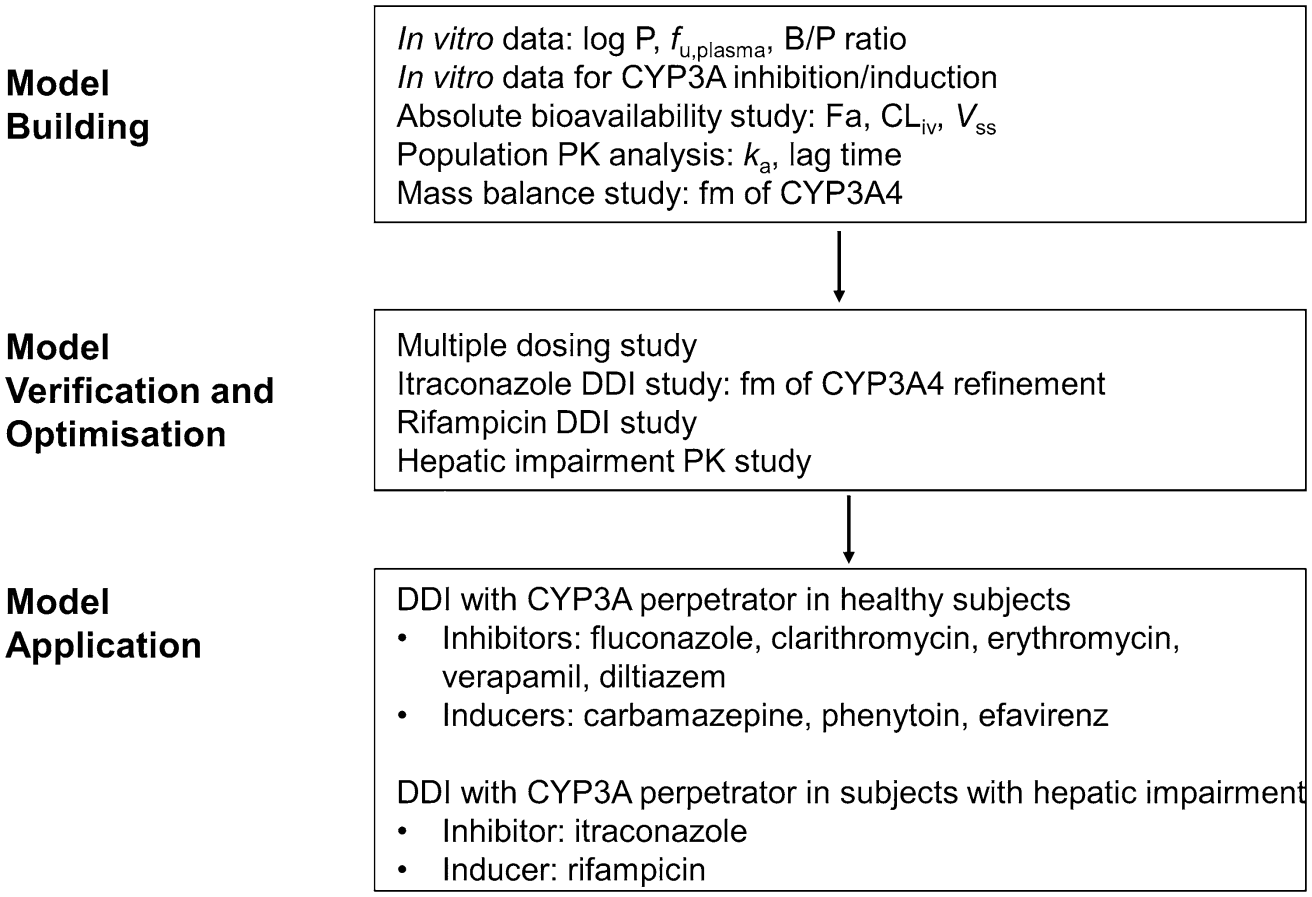
Simulation strategy. Parameters used to build and verify the model are detailed in the first two steps. The applications of the model used in this study are detailed in the final step. Abbreviations: B/P ratio, blood to plasma ratio; CL_iv,_ intravenous clearance; CYP, cytochrome P450; DDI, drug–drug interaction; Fa, absorption ratio; fm, fraction of metabolism; *f*_u,plasma_, fraction of unbound drug in plasma; *k*_*a*_, absorption rate constant; PK, pharmacokinetic; *V*_*ss*_, volume of distribution

The PK of esaxerenone was described through the implementation of the minimal PBPK model in Simcyp with the first-order absorption model. Online Resource 3 summarises model development using the stated input parameters. Figure 1 summarises the model input parameters. The log P, blood to plasma ratio (B/P ratio), fraction of unbound drug in plasma (*f*_u,plasma_), inhibition constant (*K*_*i*_), fraction of unbound drug in microsomal incubation (*f*_u,mic_), mechanism-based inhibition (MBI) maximal inactivation rate (*k*_inact_), MBI concentration at 50% *k*_inact_ (*K*_app_), and MBI *f*_u,mic_ were measured, and the maximum fold induction (Ind max) and concentration that yields half of the maximum response achievable (Ind C_50_) values were calibrated using the induction calibrator in Simcyp from measured values [8]. Values for *V*_*ss*_ and CL_iv_ were used as observed; volume of a single adjusting compartment (*V*_sac_), constant of inhibition for production response (*k*_in_), and rate constant out of a single adjusting compartment (*k*_out_) values were optimised from the observed PK profile in the bioavailability study; and the absorption ratio (Fa) value was assumed to be maximum because of the high bioavailability [6]. The values for the absorption rate constant (*k*_*a*_) and lag time were optimised and estimated, respectively, from the population PK analysis [16]. Fraction of metabolism (fm) of CYP3A4 was adjusted to reproduce the itraconazole DDI result from approximately 30% of that estimated in the mass-balance study. The fraction of drug unbound in enterocytes (*f*_u,gut_) was optimised to correspond with the itraconazole DDI result. Predicted results were compared with observed results for multiple-dosing PK, DDIs with itraconazole and rifampicin, and the effect of hepatic impairment on PK, to verify and optimise the model. Once verified, the model was used to simulate DDIs with CYP3A inhibitors (fluconazole, clarithromycin, erythromycin, verapamil, and diltiazem) and inducers (carbamazepine, phenytoin, and efavirenz) in healthy subjects and DDIs with itraconazole and rifampicin in subjects with hepatic impairment.

### Data analysis

To evaluate the predictive performance of PBPK, the Eq. (1)–(3) proposed by Guest et al. [17] were used to calculate the success criteria for maximum concentration (*C*_max_) ratio and AUC from time zero to infinity (AUC_inf_) ratio predictions. For the DDI study, the ratio was calculated as the ratio with/without the CYP3A modifier, and for the study with subjects with hepatic impairment, the ratio was subjects with/without hepatic impairment.

1 $$\text{Upper limit: }{\text{R}}_{\text{obs}} \times {\text{limit}}$$

2 $$\text{Lower limit: }{\text{R}}_{\text{obs}}/{\text{limit}}$$

3 $${\text{Limit}} \, \text{=} \, \frac{\delta \text{+2(}{\text{R}}_{\text{obs}}-1)}{{\text{R}}_{\text{obs}}}$$

*R*_obs_ represents the ratio of *C*_max_ or AUC_inf_. If the observed ratios were less than 1, the reciprocal of the ratio was used for *R*_obs_. In the present study, *δ* = 1.25 was used because the variability for esaxerenone AUC was approximately 20% of the coefficient of variation.

## Results

### Model verification and optimisation

The *k*_*a*_ was optimised to 0.8 from 0.628 as estimated by the population PK analysis to reproduce the observed PK profiles. The fm of CYP3A4 was adjusted to 0.35 to reproduce the itraconazole DDI result from approximately 0.3 as estimated in the mass-balance study. A simulated Japanese population of 27 subjects in 10 trials undergoing a 14-day regimen of 5 mg oral esaxerenone was used for multiple-dosing predictions. The simulated results compared with the observed data are shown in Online Resource 4. In the first and final 24 h studied, the observed systemic concentration matched the PBPK predicted profile. The predicted versus observed profiles of CYP3A inhibition (with itraconazole) and induction (with rifampicin) on systemic concentrations of esaxerenone, as shown in Online Resource 5 and Online Resource 6, respectively, indicated that the predicted profiles matched the observed profiles of esaxerenone in the presence of the CYP3A inhibitor, itraconazole, and the CYP3A inducer, rifampicin.

Table 1 describes the predicted DDI data compared with the observed DDI data**.** The predicted *C*_max_ and AUC_inf_ values for the control, itraconazole, and rifampicin groups were similar to those of the observed data. The predicted ratios were also comparable to the observed data and within the success criteria.

**Table 1:** Comparison of observed versus predicted pharmacokinetics in drug–drug interactions

Modifier		Control		With modifier		Ratio (with/without modifier)	
		*C*_max_ (ng/mL)	AUC_inf_ (ng/mL·h)	*C*_max_ (ng/mL)	AUC_inf_ (ng/mL·h)	*C*_max_	AUC_inf_
Itraconazole	Observed	36.4	637	41.0	976	1.13	1.53
	Predicted	30.6	673	34.6	1045	1.13	1.55
Criteria						0.85–1.51	1.01–2.31
Rifampicin	Observed	71.7	1121	47.2	350	0.66	0.31
	Predicted	59.7	1308	38.7	442	0.65	0.34
Criteria						0.44–0.99	0.18–0.55

Data are expressed as geometric means. Criteria were calculated using the equations proposed by Guest et al. [17] assuming 20% variability

*AUC*_*inf*_ area under the concentration-time curve from time zero to infinity, *C*_*max*_ maximum concentration

The predicted PK profiles of esaxerenone in subjects with hepatic impairment were compared with the observed data. The Simcyp population used for subjects with hepatic impairment was based on a Caucasian population, but the clinical hepatic impairment study in comparison was performed in Japanese subjects. It was observed that the PK profiles of esaxerenone were similar for both Japanese and Caucasian subjects. Thus, the predictive performance of the ethnic difference was first evaluated, and the results of this analysis are summarised in Online Resource 7. The predicted results reproduced the observed data which found the PK parameters of esaxerenone to be similar in Japanese and Caucasian populations, allowing for comparisons to be made between the observed and predicted PK data for esaxerenone in subjects with hepatic impairment. Table 2 shows the predicted versus observed PK parameters of esaxerenone in subjects with hepatic impairment. The predicted and observed *C*_max_ and AUC_inf_ values were similar for normal age-matched subjects and for subjects with mild (CP-A) and moderate (CP-B) hepatic impairment. However, the hepatically impaired to normal exposure ratio, although within the success criteria, was slightly over-predicted compared with the observed data.

**Table 2:** Comparison of the observed versus predicted pharmacokinetics of esaxerenone in hepatic impairment

Population		PK parameter		Ratio (HI/Normal)	
		*C*_max_ (ng/mL)	AUC_inf_ (ng·h/mL)	*C*_max_	AUC_inf_
Normal	Observed	25.8	608	NA	NA
	Predicted	25.3	655	NA	NA
Mild	Observed	24.8	501	0.96	0.82
	Predicted	23.8	704	0.94	1.07
	Criteria			0.75–1.23	0.59–1.14
Moderate	Observed	20.8	667	0.80	1.10
	Predicted	23.9	868	0.94	1.33
	Criteria			0.57–1.12	0.83–1.45

Data are expressed as geometric means. Criteria were calculated using the equations proposed by Guest et al. [17] and assuming 20% variability

*AUC*_*inf*_ area under the concentration-time curve from time zero to infinity, *C*_*max*_ maximum concentration, *HI* hepatic impairment, *NA* not applicable, *PK* pharmacokinetic

### Model application

**Fig. 2:**
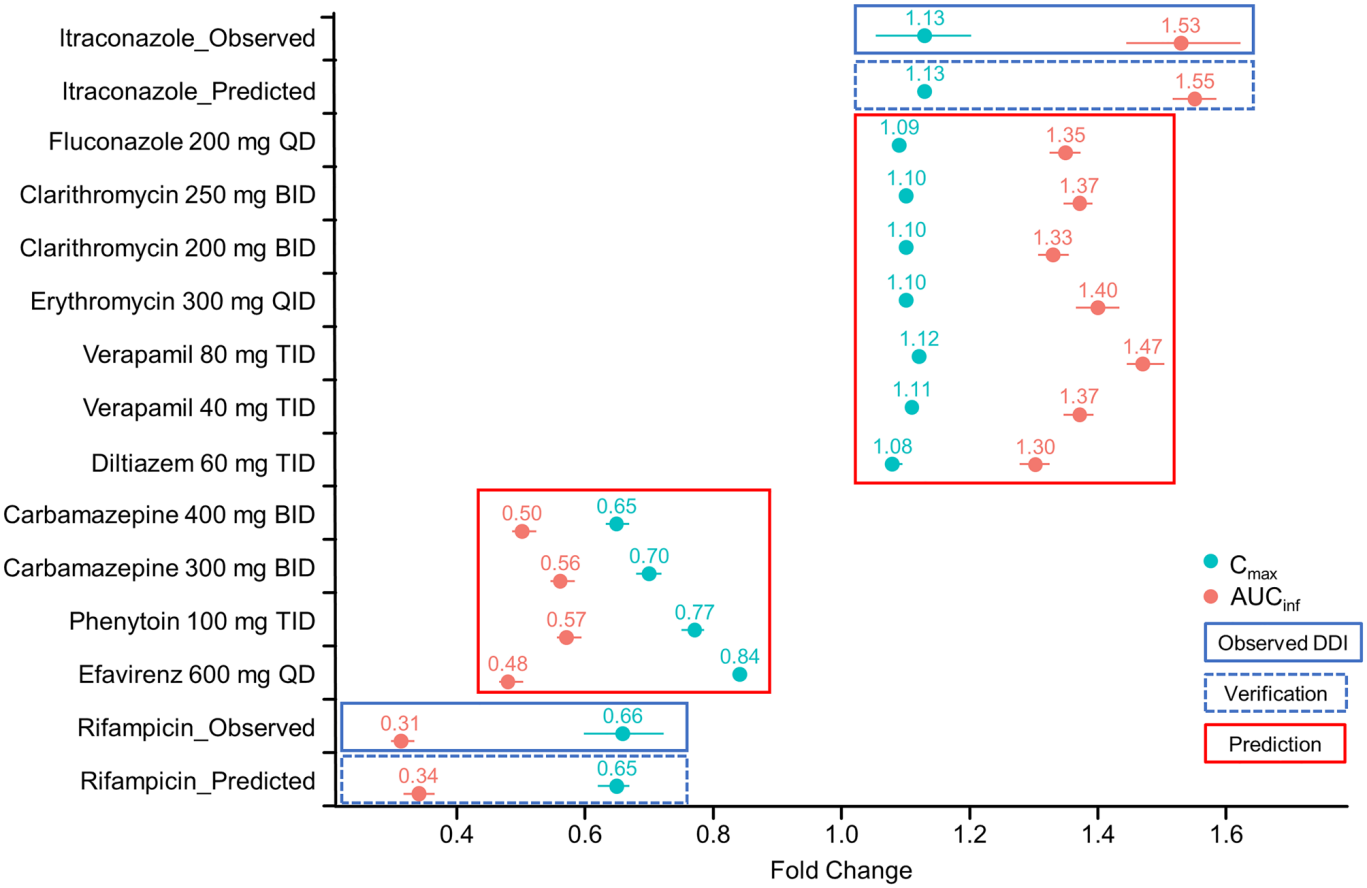
Predicted impact of CYP3A inhibitor/inducer on the pharmacokinetics of esaxerenone in healthy subjects. Esaxerenone was administered on day 6 at 2.5 mg when administered with inhibitors and at 5 mg when administered with inducers. The modifiers were administered for 9 days. Bars represent the 90% confidence interval. The fold change represents the ratio (with/without modifier). Abbreviations: AUC_inf_, area under the concentration-time curve from time zero to infinity; BID, twice daily; *C*_max_, maximum concentration; CYP, cytochrome P450; DDI, drug–drug interaction; QD, once daily; QID, four times a day; TID, three times a day

The PBPK model predicted the effect of untested CYP3A modifiers on the plasma exposure of esaxerenone in healthy subjects as shown in Figure 2. For CYP3A inhibitors, *C*_max_-fold changes ranged from 1.08 to 1.12 and AUC_inf_-fold changes ranged from 1.30 to 1.47. For CYP3A inducers, *C*_max_ fold changes ranged from 0.48 to 0.57 and AUC_inf_-fold changes ranged from 0.65 to 0.84. The predicted fold changes of AUC_inf_ and *C*_max_ with these CYP3A inhibitors were less than those with itraconazole, except in the case of verapamil, in which the predicted AUC_inf_ increase was close to that of itraconazole. The effect of these CYP3A inducers on esaxerenone PK was predicted to be weaker than rifampicin.

Table 3 describes the predicted DDIs with itraconazole and rifampicin in subjects with normal hepatic function and subjects with mild to moderate hepatic impairment according to the PBPK model. Ratios of *C*_max_ and AUC_inf_ with and without the modifier were calculated for subjects with normal hepatic function as well as subjects with mild to moderate hepatic impairment. In addition, the ratios of *C*_max_ and AUC_inf_ were compared between subjects with normal hepatic function and subjects with mild to moderate hepatic impairment. The effect of the CYP3A inhibitor and inducer tended to weaken as the severity of hepatic impairment increased.

**Table 3:** Predicted drug–drug interactions in patients with normal hepatic function and mild to moderate hepatic impairment

Modifier		Normal		Mild		Moderate	
		*C*_max_	AUC_inf_	*C*_max_	AUC_inf_	*C*_max_	AUC_inf_
Itraconazole	Ratio (with/without modifier)	1.15	1.69	1.14	1.61	1.08	1.33
	Ratio (HI/Normal)	NA	NA	0.99	0.95	0.94	0.79
Rifampicin	Ratio (with/without modifier)	0.59	0.28	0.61	0.29	0.72	0.40
	Ratio (HI/Normal)	NA	NA	1.03	1.04	1.22	1.43

Data are expressed as geometric means

*AUC*_*inf*_ area under the concentration-time curve from time zero to infinity, *C*_*max*_ maximum concentration, *HI* hepatic impairment, *NA* not applicable

## Discussion

In this study, a PBPK model was developed to predict the effect of CYP3A modifiers on esaxerenone PK in healthy subjects and subjects with hepatic impairment. Such data can help inform the appropriate use of esaxerenone in the clinical setting. Of note, accurate prediction models are difficult to build, especially in subjects with functional impairments such as those with hepatic impairment, because parameters vary between each functional impairment; therefore, some level of deviation is expected. However, DDI prediction models are important for these specific functional impairment subpopulations because clinical trials within these groups are difficult to conduct.

The PBPK model constructed in this study used in vitro data, as well as the results of mass-balance [7] and bioavailability [6] studies. Some parameters in the model required adjustment, such as the contribution of CYP3A metabolism, which was performed using the results from the DDI study with itraconazole [9].

Previous in vitro studies showed that esaxerenone has both inhibitory and inducing potentials for CYP3A [8]. Because these effects counteract one another, esaxerenone has a low potential for DDIs as a perpetrator overall. Specifically, administration of 5 mg/day esaxerenone for 14 days produced an approximately 1.2-fold increase in the AUC of midazolam as a CYP3A index substrate [15]. However, because esaxerenone might inhibit or induce its own metabolism by CYP3A, these parameters for CYP3A inhibition/induction were also incorporated into our PBPK model.

To assess the predictive performance for DDIs of the PBPK model, the success criteria for *C*_max_ and AUC ratios were calculated using the equations proposed by Guest et al., which tend to introduce bias at lower interaction levels compared with the 2-fold criterion used conventionally [17] because the observed fold changes for *C*_max_ and AUC of esaxerenone with CYP3A modifiers are not as comparatively high. This approach of incorporating the equations by Guest et al. is encouraged when evaluating the accuracy of model predictions for DDIs [18].

The PBPK model in the current study successfully reproduced the results of a clinical multiple-dosing study. In addition, the PBPK model successfully predicted a DDI study with itraconazole and rifampicin in which itraconazole increased the AUC_inf_ of esaxerenone by 53.1%, whereas rifampicin reduced it by 68.8% [9].

Prior to hepatic impairment predictions, evaluation of PK differences in Japanese versus Caucasian ethnicities was performed. The Simcyp built-in population files for hepatic impairment are modelled from Caucasian subject data [19]; however, the esaxerenone hepatic impairment study being used for comparison was performed in Japanese subjects [10]. It has been reported and incorporated in the Simcyp built-in population file for Japanese subjects that liver volume and hepatic CYP3A4 abundance, which are considered to affect the PK of esaxerenone, are lower in Japanese subjects than in Caucasian subjects [20]. The PBPK model successfully reproduced the observed data, indicating that esaxerenone PK parameters are similar in Japanese and Caucasian populations. This enables the model to be used to compare the hepatic impairment study with that of the PBPK predictions.

In the study by Kurata et al., it was observed that hepatic impairment had no clinically relevant effect on esaxerenone exposure [6], and similar results were found with the PBPK prediction. The PBPK model successfully predicted PK parameters within the success criteria; however, there was a tendency to overpredict the exposure ratios compared with the observed data. This is consistent with a previous study [21] where it was observed that PBPK modelling tended to overpredict the AUC changes of low clearance compounds in subjects with hepatic impairment. Changes in physiological parameters such as albumin concentration, haematocrit, cardiac output, blood flow, and the expression and activities of metabolic enzymes and transporters have been incorporated into the hepatic impairment population file based on published findings [19]. The study also showed that changes in absorption in patients with hepatic impairment might be important. Reduced bile flow or lower bile acid concentration might lead to a reduced drug absorption. Such changes in absorption in subjects with hepatic impairment were not considered in this PBPK model; however, the reduction in absorption may contribute to the less than unity AUC ratio observed in the mild hepatic impairment data. This is a limitation of the current PBPK model.

Esaxerenone is metabolised by UGT in addition to CYP3A [7]. Rifampicin has an inductive effect on UGTs as well as CYP3A [9]. When incorporating the inductive effect of rifampicin on UGTs into the PBPK model, the effect of rifampicin on esaxerenone PK might be more strongly predicted. However, this could not be performed in the current study because the contribution of UGT on the metabolism of esaxerenone has not been clarified. This is another limitation of the current PBPK model.

This PBPK model was applied to predict the DDIs of esaxerenone with CYP3A modulators that were not clinically tested. The effects of untested CYP3A inhibitors and inducers on the PK of esaxerenone in healthy subjects were predicted with the PBPK model**.**

According to the package insert in Japan, esaxerenone requires caution when being coadministered with strong CYP3A inhibitors or inducers. The AUC changes of esaxerenone with clarithromycin (a strong CYP3A inhibitor), and fluconazole, erythromycin, and diltiazem (moderate CYP3A inhibitors) were predicted to be less than that with itraconazole, except in the case of verapamil (a moderate CYP3A inhibitor), in which the predicted AUC increase with verapamil was close to that seen with itraconazole. A DDI study has been published reporting verapamil with midazolam as a clinical index substrate for CYP3A, in which 80 mg of verapamil was orally administered three times daily for 2 days to healthy subjects, and 15 mg of midazolam was orally administered on the second day [22]. As the AUC of midazolam increased 2.9-fold, this result led to verapamil being classified as a moderate CYP3A inhibitor. However, the dosing duration of 2 days for verapamil used in this study appears too short to fully inhibit CYP3A. The predicted AUC increase of midazolam was 7.0-fold when verapamil was administered in the same scenario as this study (80 mg of oral verapamil three times daily for 9 days and 15 mg of oral midazolam on day 6) (Online Resource 8). From this result, verapamil could be considered a strong CYP3A inhibitor. Potassium levels should be carefully monitored when esaxerenone is co-administered with inhibitors and the dose should be adjusted if needed. The AUC changes of esaxerenone with carbamazepine and phenytoin (strong CYP3A inducers) and efavirenz (a moderate CYP3A inducer) were predicted to be less than that with rifampicin.

The effects of strong CYP3A modulators on the PK of esaxerenone in subjects with hepatic impairment were predicted with the current PBPK model. The effects of CYP3A inhibitors or inducers tended to weaken as the severity of hepatic impairment increased. A decrease in CYP3A abundance dependant on the severity of hepatic impairment has been incorporated into the hepatic impairment population file [19]. As a result, the contribution of CYP3A to the metabolism of esaxerenone decreased as the severity of hepatic impairment increased. The effect of CYP3A modulators on the esaxerenone PK in subjects with hepatic impairment may be similar or weaker than in subjects with normal hepatic function. It can therefore be concluded that, for concomitant use of CYP3A modulators, caution is advised for subjects with hepatic impairment, as is for healthy subjects.

In conclusion, the effects of the moderate inhibitors and inducers were lower than those of the strong CYP3A inhibitors and inducers, and for all subjects (including healthy subjects, as well as those with hepatic impairment), caution is needed when there is concomitant use of CYP3A modulators. By using this PBPK model, DDIs were predicted without the need for conducting clinical trials, contributing to providing information for the appropriate use of esaxerenone.

## Supplementary Information

Below is the link to the electronic supplementary material.

Supplementary file1 (DOCX 239 KB)

## Data Availability

Clinical data used for the model development are sourced from the published articles that are cited accordingly in this manuscript. Output data obtained in this study are available within the article and its supplementary materials.
