# Supplementary material for: Physiologically based pharmacokinetic modelling to predict the clinical effect of CYP3A inhibitors/inducers on esaxerenone pharmacokinetics in healthy subjects and subjects with hepatic impairment
Source: Eur J Clin Pharmacol. 2021 Aug 20;78(1):65–73. doi: 10.1007/s00228-021-03194-x (PMC8724184; doi:10.1007/s00228-021-03194-x)
Supplement: Supplementary file 1 — Supplementary file1 (DOCX 239 KB) [file 228_2021_3194_MOESM1_ESM.docx]

# Physiologically Based Pharmacokinetic Modelling to Predict the Clinical Effect of CYP3A Inhibitors/Inducers on Esaxerenone Pharmacokinetics in Healthy Subjects and Subjects with Hepatic Impairment

***Supplementary materials***

***European Journal of Clinical Pharmacology***

Akiko Watanabe,^1^ Tomoko Ishizuka,^2^ Makiko Yamada,^2^ Yoshiyuki Igawa,^1^ Takako Shimizu,^1^ Hitoshi Ishizuka^1^

^1^Quantitative Clinical Pharmacology Department, Daiichi Sankyo Co., Ltd., Tokyo, Japan

^2^Drug Metabolism and Pharmacokinetics Research Laboratories, Daiichi Sankyo Co., Ltd., Tokyo, Japan

***Corresponding author***

Akiko Watanabe

Address: Quantitative Clinical Pharmacology Department, Daiichi Sankyo Co., Ltd.,

1-2-58 Hiromachi Shinagawa-ku Tokyo, Japan

Tel: +81-3-3492-3131

Fax: +81-3-5436-8567

E-mail: watanabe.akiko.mi@daiichisankyo.co.jp

**Online Resource 1. Chemical structure of esaxerenone**

**Online Resource 2. Trial designs for simulations**

| **Trial** | **Population** | **Sample size** | **Age (years)** | **Dosing regimen for esaxerenone** | **Dosing regimen for modifier** | **Clinical study**  **Ref.** |
| --- | --- | --- | --- | --- | --- | --- |
| Multiple dosing in HV | Sim-Japanese | 27 subjects × 10 trials | 20–45 | Oral 5 mg QD for 14 days | NA | [15] |
| DDI with itraconazole in HV | Sim-Japanese | 20 subjects × 10 trials | 20–45 | Oral 2.5 mg on day 6 | Oral 200 mg BID on day 1, QD from day 2 to 9 | [9] |
| DDI with rifampicin in HV | Sim-Japanese | 11 subjects × 10 trials | 20–45 | Oral 5 mg on day 6 | Oral 600 mg QD for 9 days | [9] |
| DDI with fluconazole in HV | Sim-Japanese | 20 subjects × 10 trials | 20–45 | Oral 2.5 mg on day 6 | Oral 200 mg QD for 9 days | NA |
| DDI with clarithromycin in HV | Sim-Japanese | 20 subjects × 10 trials | 20–45 | Oral 2.5 mg on day 6 | Oral 200 or 250 mg BID for 9 days | NA |
| DDI with erythromycin in HV | Sim-Japanese | 20 subjects × 10 trials | 20–45 | Oral 2.5 mg on day 6 | Oral 300 mg QID for 9 days | NA |
| DDI with verapamil in HV | Sim-Japanese | 20 subjects × 10 trials | 20–45 | Oral 2.5 mg on day 6 | Oral 40 or 80 mg TID for 9 days | NA |
| DDI with diltiazem in HV | Sim-Japanese | 20 subjects × 10 trials | 20–45 | Oral 2.5 mg on day 6 | Oral 60 mg TID for 9 days | NA |
| DDI with carbamazepine in HV | Sim-Japanese | 20 subjects × 10 trials | 20–45 | Oral 5 mg on day 6 | Oral 300 or 400 mg BID for 9 days | NA |
| DDI with phenytoin in HV | Sim-Japanese | 20 subjects × 10 trials | 20–45 | Oral 5 mg on day 6 | Oral 100 mg TID for 9 days | NA |
| DDI with efavirenz in HV | Sim-Japanese | 20 subjects × 10 trials | 20–45 | Oral 5 mg on day 6 | Oral 600 mg QD for 9 days | NA |
| Single dosing in Japanese HV (ethnic difference PK) | Sim-Japanese | 12 subjects × 10 trials | 20–45 | Oral 20 mg | NA | NA |
| Single dosing in Caucasian HV (ethnic difference PK) | Sim-Healthy Volunteers | 12 subjects × 10 trials | 20–45 | Oral 20 mg | NA | NA |
| Single dosing in normal HI | Sim-Healthy Volunteers, age-matched for HI* | 20 subjects × 10 trials | 20–65 | Oral 2.5 mg | NA | [10] |
| Single dosing in mild HI | Sim-Cirrhosis CP-A | 20 subjects × 10 trials | 20–65 | Oral 2.5 mg | NA | [10] |
| Single dosing in moderate HI | Sim-Cirrhosis CP-B | 20 subjects × 10 trials | 20–65 | Oral 2.5 mg | NA | [10] |
| DDI with itraconazole in normal HI | Sim-Healthy Volunteers, age-matched for HI* | 20 subjects × 10 trials | 20–65 | Oral 2.5 mg on day 6 | Oral 200 mg BID on day 1, QD from day 2 to 9 | NA |
| DDI with itraconazole in mild HI | Sim-Cirrhosis CP-A | 20 subjects × 10 trials | 20–65 | Oral 2.5 mg on day 6 | Oral 200 mg BID on day 1, QD from day 2 to 9 | NA |
| DDI with itraconazole in moderate HI | Sim-Cirrhosis CP-B | 20 subjects × 10 trials | 20–65 | Oral 2.5 mg on day 6 | Oral 200 mg BID on Day 1, QD from day 2 to 9 | NA |
| DDI with rifampicin in normal HI | Sim-Healthy Volunteers, age-matched for HI* | 20 subjects × 10 trials | 20–65 | Oral 2.5 mg on day 6 | Oral 600 mg QD for 9 days | NA |
| DDI with rifampicin in mild HI | Sim-Cirrhosis CP-A | 20 subjects × 10 trials | 20–65 | Oral 2.5 mg on day 6 | Oral 600 mg QD for 9 days | NA |
| DDI with rifampicin in moderate HI | Sim-Cirrhosis CP-B | 20 subjects × 10 trials | 20–65 | Oral 2.5 mg on day 6 | Oral 600 mg QD for 9 days | NA |

*Distribution of ages in Sim-Healthy Volunteers was matched to that in Sim-Cirrhosis CP-A and CP-B.

Abbreviations: BID, twice daily; CP-A, Child–Pugh scores for mild level of cirrhosis, score level A; CP-B, Child–Pugh scores for moderate level of cirrhosis, score level B; DDI; Drug–drug interaction; HI, hepatic impairment; HV, healthy volunteers; NA, not applicable; PK, pharmacokinetics; QD, once daily; QID, four times a day; TID, three times a day.

**Online Resource 3. Input parameters**

| **Input parameter** | **Value** | **Source** |
| --- | --- | --- |
| MW | 466.47 |  |
| Compound type | Neutral |  |
| Log P | 3.4 | Measured [8] |
| B/P ratio | 0.758 | Measured [8] |
| *f*_u,plasma_ | 0.01 | Measured [8] |
| Fa | 1 (5% CV) | Assumed maximum value due to high BA (approximately 90% in absolute BA study) [10] |
| *k*_a_ (1/h) | 0.8 (optimised) | Optimised from 0.628 estimated by population PK [16] |
| Lag time (h) | 0.444 | Estimated by population PK [16] |
| *f*_u,gut_ | 0.85 (optimised) | Optimised to recover itraconazole DDI result |
| *Q*_gut_ | 11.794 | Predicted in Simcyp |
| *V*_ss_ (L/kg) | 1.34 (11.8% CV) | Observed in absolute BA study [10] |
| *V*_sac_ (L/kg) | 0.81 | Optimised from absolute BA study [10] |
| *k*_in_ (1/h) | 0.71 | Optimised from absolute BA study |
| *k*_out_ (1/h) | 0.27 | Optimised from absolute BA study [10] |
| *K*_p,liver:plasma_ | 10 | Referred to observed rat *K_p,liver:plasma_* |
| CL_iv_ (L/h) | 3.69 | Observed in absolute BA study [10] |
| fm of CYP3A4 | 0.35 | Adjusted to reproduce itraconazole DDI result from approximately 30% estimated in mass balance study [7] |
| CYP3A4 CL_int_ (μL/min/pmol) | 0.310 | Back-calculated from CL_iv_ and fm of CYP3A4 using the retrograde model in Simcyp |
| Interaction with CYP3A4 |  |  |
| *K*_i_ (µM) | 40.3 | Measured [8] |
| *f*_u,mic_ | 0.434 | Measured at 0.2 mg/mL [8] |
| MBI *K*_app_ (µM) | 44.8 | Measured [8] |
| MBI *k*_inact_ (1/h) | 1.41 | Measured [8] |
| MBI *f*_u,mic_ | 0.066 | Measured at 2 mg/mL [8] |
| Ind max | 11.8 | Calibrated from the measured value [8] using the induction calibrator in Simcyp |
| Ind C_50_ (µM) | 2.96 | Calibrated from the measured value [8] using the induction calibrator in Simcyp |
| *f*_u,inc_ | 1 | Default in Simcyp |
| *γ* | 1 | Default in Simcyp |

Abbreviations: BA, bioavailability; B/P ratio, blood to plasma ratio; CL_iv,_ intravenous clearance; CL_int_, intrinsic clearance; CV, coefficient of variation; CYP, cytochrome P450; DDI, drug–drug interaction; *F*a, absorption ratio; fm, fraction of metabolism; *f*_u,inc_, fraction of unbound drug in the in vitro incubation; *f*_u,mic,_ fraction of unbound drug in microsomal incubation; *f*_u,plasma_, fraction of unbound drug in plasma; *f*_u,gut_, fraction of unbound drug in enterocytes; Ind max, maximum fold induction; Ind C_50,_ concentration that yields half of the maximum response achievable; *k*_a_, absorption rate constant; *K*_i_, inhibition constant; *k*_in_, rate constant into a single adjusting compartment; *K*_app_, concentration of mechanism-based inhibitor associated with half maximal inactivation rate; *k*_inact,_ maximal inactivation rate; *k*_out,_ rate constant out of a single adjusting compartment; *K*_p,liver:plasma_, liver:plasma partition coefficient; MBI, mechanism-based inhibition; MW, molecular weight; PK, pharmacokinetic; pop, population; *Q*_gut_, nominal flow through the gut; *V*_sac_, volume of a single adjusting compartment; *V*_ss_, steady state volume of distribution.

**Online Resource 4. Observed and predicted pharmacokinetic profiles of esaxerenone after multiple oral dosing of esaxerenone in healthy subjects**

Top panel; linear, Bottom panel; semi-log. Esaxerenone 5 mg was administered once daily for 14 days.

Abbreviation: CSys, systemic concentration.

**Online Resource 5. Observed and predicted pharmacokinetic profiles of esaxerenone after single oral dosing of esaxerenone with and without itraconazole in healthy subjects**

Top panel; linear, Bottom panel; semi-log. Itraconazole 200 mg was administered twice a day on day 1, followed by once daily on days 2–9. Esaxerenone was administered at 2.5 mg on day 6.

Abbreviations: CSys, systemic concentration; CSysl, systemic concentration with an inhibitor or an inducer.

**Online Resource 6. Observed and predicted pharmacokinetic profiles of esaxerenone after single oral dosing of esaxerenone with and without rifampicin in healthy subjects**

Top panel; linear, Bottom panel; semi-log. Rifampicin 600 mg was administered once daily for 9 days and 5 mg of esaxerenone was administered on day 6.

Abbreviations: CSys, systemic concentration; CSysl, systemic concentration with an inhibitor or an inducer.

**Online Resource 7. Predictability of the impact of ethnicity on the pharmacokinetics of esaxerenone**

| **Population** |  | **PK parameter** | | **Ratio (Caucasian/Japanese)** | |
| --- | --- | --- | --- | --- | --- |
|  |  | *C*_max_  (ng/mL) | AUC_inf_  (ng·h/mL) | *C*_max_ | AUC_inf_ |
| Japanese | Observed | 228 | 4331 | NA | NA |
|  | Predicted | 248 | 5441 | NA | NA |
| Caucasian | Observed | 211 | 4076 | 0.93 | 0.94 |
|  | Predicted | 197 | 4414 | 0.79 | 0.81 |
|  | Criteria |  |  | 0.71–1.21 | 0.73–1.22 |

Data are expressed as geometric means. Criteria were calculated using the equations proposed by Guest et al. [17] and assuming 20% variability.

Abbreviations: AUC_inf_, area under the concentration-time curve from time zero to infinity; *C*_max_, maximum concentration; NA, not applicable; PK, pharmacokinetic.

**Online Resource 8. Prediction of the effect of verapamil on midazolam pharmacokinetics**

**Trial design**

| **Population** | **Sample size** | **Age (years)** | **Dosing regimen for midazolam** | **Dosing regimen for modifier** |
| --- | --- | --- | --- | --- |
| Sim-Japanese | 20 subjects × 10 trials | 20–45 | Oral 15 mg on day 6 | Oral 80 mg TID for 9 days |

**Predicted DDI**

| **Control** | | **With modifier** | | **Ratio (with/without modifier)** | |
| --- | --- | --- | --- | --- | --- |
| *C*_max_ (ng/mL) | AUC_inf_ (ng/mL·h) | *C*_max_ (ng/mL) | AUC_inf_ (ng/mL·h) | *C*_max_ | AUC_inf_ |
| 71.5 | 205 | 193 | 1457 | 2.70 | 7.12 |

Abbreviations: AUC_inf_, area under the concentration-time curve from time zero to infinity; *C*_max_, maximum concentration; DDI, drug–drug interaction; TID, three times daily.
